# Placental Growth Factor Contributes to Liver Inflammation, Angiogenesis, Fibrosis in Mice by Promoting Hepatic Macrophage Recruitment and Activation

**DOI:** 10.3389/fimmu.2017.00801

**Published:** 2017-07-11

**Authors:** Xi Li, Qianwen Jin, Qunyan Yao, Yi Zhou, Yanting Zou, Zheng Li, Shuncai Zhang, Chuantao Tu

**Affiliations:** ^1^Department of Geriatrics, Zhongshan Hospital, Fudan University, Shanghai, China; ^2^Department of Gastroenterology and Hepatology, Zhongshan Hospital, Fudan University, Shanghai, China; ^3^Shanghai Institute of Liver Diseases, Shanghai, China; ^4^Laboratory Animal Center, Zhongshan Hospital, Fudan University, Shanghai, China

**Keywords:** placental growth factor, hepatic fibrosis, inflammation, macrophage, Kupffer cells, hepatic stellate cells, angiogenesis, small interfering RNA

## Abstract

Placental growth factor (PlGF), a member of the vascular endothelial growth factor (VEGF) family, mediates wound healing and inflammatory responses, exerting an effect on liver fibrosis and angiogenesis; however, the precise mechanism remains unclear. The aims of this study are to identify the role of PlGF in liver inflammation and fibrosis induced by bile duct ligation (BDL) in mice and to reveal the underlying molecular mechanism. PlGF small interfering RNA (siRNA) or non-targeting control siRNA was injected by tail vein starting 2 days after BDL. Liver inflammation, fibrosis, angiogenesis, macrophage infiltration, and hepatic stellate cells (HSCs) activation were examined. Our results showed that PlGF was highly expressed in fibrotic livers and mainly distributed in activated HSCs and macrophages. Furthermore, PlGF silencing strongly reduced the severity of liver inflammation and fibrosis, and inhibited the activation of HSCs. Remarkably, PlGF silencing also attenuated BDL-induced hepatic angiogenesis, as evidenced by attenuated liver endothelial cell markers CD31 and von Willebrand factor immunostaining and genes or protein expression. Interestingly, these pathological ameliorations by PlGF silencing were due to a marked reduction in the numbers of intrahepatic F4/80^+^, CD68^+^, and Ly6C^+^ cell populations, which were reflected by a lower expression of these macrophage marker molecules in fibrotic livers. In addition, knockdown of PlGF by siRNA inhibited macrophages activation and substantially suppressed the expression of pro-inflammatory cytokines and chemokines in fibrotic livers. Mechanistically, evaluation of cultured RAW 264.7 cells revealed that VEGF receptor 1 (VEGFR1) mainly involved in mediating the role of PlGF in macrophages recruitment and activation, since using VEGFR1 neutralizing antibody blocking PlGF/VEGFR1 signaling axis significantly inhibited macrophages migration and inflammatory responses. Together, these findings indicate that PlGF plays an important role in liver inflammation, angiogenesis, and fibrosis by promoting hepatic macrophage recruitment and activation, and suggest that blockage of PlGF could be a promising novel therapy for chronic fibrotic liver diseases.

## Introduction

Liver fibrosis is the final common pathway of chronic liver diseases of various etiologies, which develops as a result of the sustained wound-healing process triggered by liver injury and inflammation (1–3). As chronic liver injury process, hepatic stellate cells (HSCs) become activated and transdifferentiate to myofibroblast-like cells, leading to the excess accumulation of extracellular matrix (ECM) (1–4). It has been well established that HSC activation results from the inflammatory activity of liver immune cells, predominantly macrophages (2–7). Furthermore, activated myofibroblasts can also amplify inflammatory responses by inducing the infiltration of macrophages and further secreting cytokines (4, 5). Consequently, understanding the mechanism of inflammation and fibrosis is critically important to develop treatments for chronic liver diseases (2).

Recent studies in animal models and in cirrhotic patients have provided key insights regarding the role of liver macrophages in regulating hepatic fibrogenesis and fibrosis regression (4–13). Hepatic macrophages can arise not only from proliferating resident macrophages but also from circulating monocyte that originates in the bone marrow (BM), which are recruited to the injured liver (4–6). In addition, these cells have been classified either into “pro-inflammatory” M1 or “immunoregulatory” M2 macrophages, though such binary classifications cannot represent the complex *in vivo* environment for most macrophage subsets (6, 7, 12, 13). Upon liver injury, macrophages activate and produce cytokines (TGF-β, TNF-α, and interleukin-1β), and chemokines, such as CC-chemokine ligand 2 (CCL2, also MCP-1), CCL5 (RANTES), and CXCL10 (2–8). In addition, HSCs may directly recruit Kupffer cells and circulating macrophages by the expression of adhesion molecules, such as intercellular adhesion molecule 1 (ICAM-1), vascular cell adhesion molecular-1 (VCAM-1), and E-selection (2). Therefore, chemokines and adhesion also play a pivotal role in the recruitment and differentiate of monocyte and macrophages to the sites of inflammation through receptors among the inflammatory mediators (7–10); leading to the development and progression of liver injury, inflammation, and fibrosis (3–12). Macrophages also can release large amounts of angiogenic cytokine vascular endothelial growth factor (VEGF) and induce the formation of new blood vessel growth during wound repair, inflammation, and tumor growth (9, 12–16). However, the mechanisms modulating chemokine pathways and hepatic macrophages in liver fibrogenesis are not fully understood (2–5). Therefore, elucidating the complex regulatory mechanisms by which macrophages promote inflammation and fibrosis might lead to novel therapies to suppress liver inflammation and prevent the development fibrosis (4, 5, 12).

Placental growth factor (PlGF), a member of the VEGF family, is a pleiotropic cytokine that stimulates endothelial cell (EC) growth, migration, and survival; chemoattracts macrophages and BM progenitors; and promotes pathologic angiogenesis and wound healing (17–22). Unlike VEGF, PlGF selectively binds VEGF receptor 1 (VEGFR1) and its coreceptors neurophilin-1 and -2 (17, 18). It is noteworthy that PlGF is dispensable for development and health, while blockage of PlGF pathway has been shown to reduce pathological angiogenesis without affecting healthy blood vessels (17, 18, 21). Recent reports have demonstrated that PlGF is overexpressed in cirrhotic liver and hepatocellular carcinoma (HCC) both in human and in rodent models (18, 22–26). Furthermore, we and others previously have shown that blockade of PlGF by specific antibody, small interfering RNA (siRNA), or genetic ablation suppressed liver fibrogenesis (22, 23), reduced portal hypertension (24) and inhibited HCC (18, 25, 26). Thus, PlGF signaling represents a promising target for therapy of chronic liver disease with angiogenesis (17, 22–26).

However, the mechanism underlying PlGF mediates the pathogenesis of liver fibrosis has not been fully elucidated, and identifying the novel pathological role of PlGF is very important for clinical translational research. Therefore, the aims of the study were to identify the role for PlGF in mediating liver inflammation and fibrosis and to reveal the mechanistic links of PlGF signaling between hepatic macrophages recruitment, inflammatory response, and HSC activation in the context of the fibrotic liver microenvironment.

## Materials and Methods

### Chemicals and Reagents

Lipopolysaccharide (LPS), Sirius red F3B, and saturated aqueous solution of picric acid were from Sigma Chemical, Co. Ltd. (St. Louis, MO, USA). Fetal bovine serum (FBS), trypsin, Dulbecco’s modified Eagle medium (DMEM), penicillin, and streptomycin were from Gibco (Carlsbad, CA, USA). Invivofectamine^®^ 2.0 reagent, *in vivo* predesigned PlGF siRNA and *in vivo* non-targeting control (NTC) siRNA were from Life Technologies (Carlsbad, CA, USA). siRNA sequences are provided in the supporting information (Figure S1 in Supplementary Material). Recombinant mouse PlGF-2 protein was from R&D Systems Inc. (Minneapolis, MN, USA).

### Animals and Experimental Design

**Figure 1:**
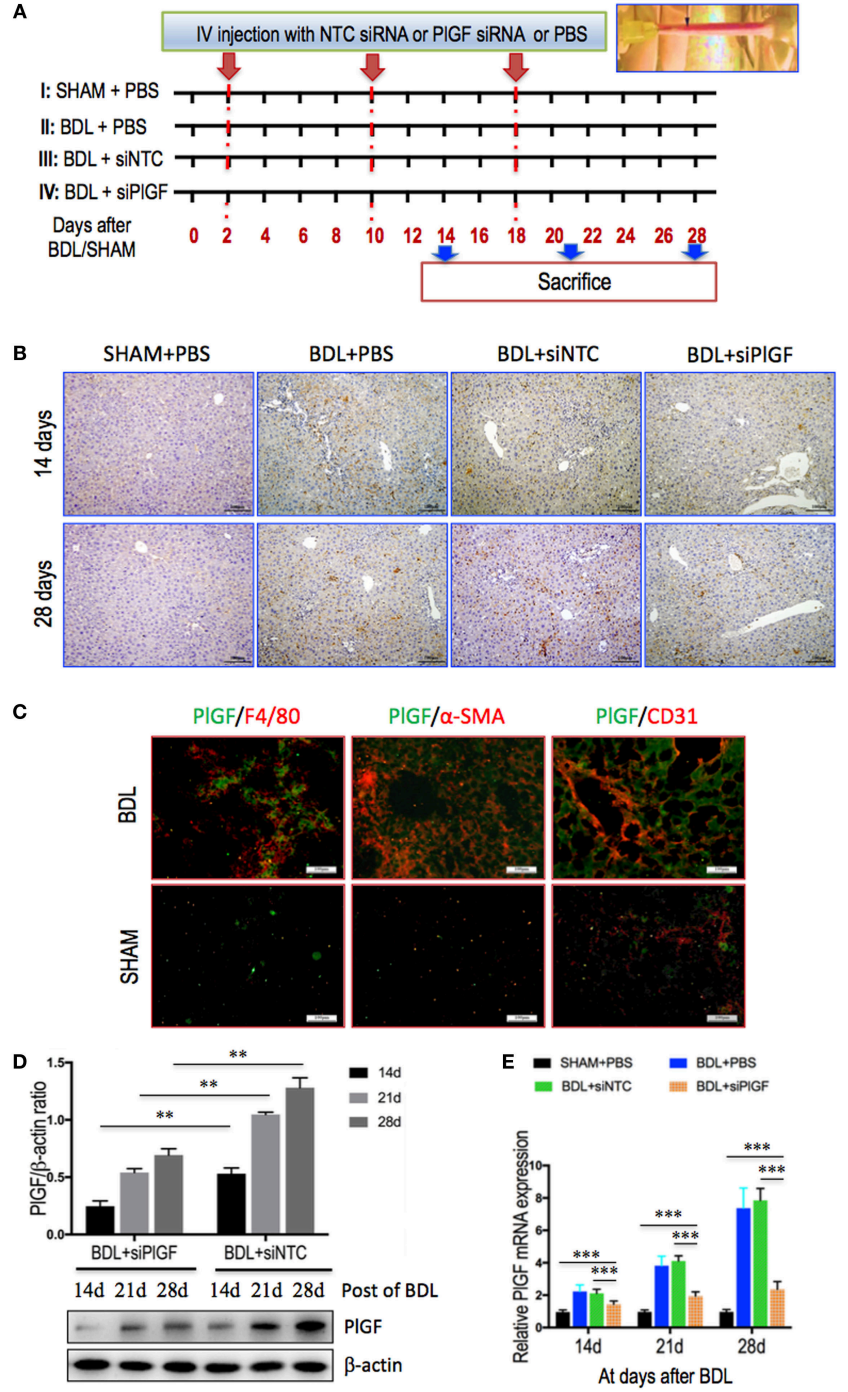
Placental growth factor (PlGF) is highly induced in fibrotic liver and PlGF silencing robustly limits intrahepatic PlGF overexpression in bile duct ligation (BDL) mice. **(A)** Experimental study design. Mice were induced liver fibrosis by BDL for 28 days and treated with PlGF siRNA (siPlGF) or non-targeting control siRNA (siNTC) or PBS *via* tail vein injection (IV) at days 2, 10, and 18 after BDL. Six to ten mice of each group were sacrificed on days 14, 21, and 28 after BDL, respectively. Control mice were sham-operated (SHAM). **(B)** Representative microscopy images PlGF immunohistochemistry in livers from each group at days 14 and 28 after BDL or sham-operated (SHAM). Original magnification: 100×. **(C)** Immunofluorescent double staining in liver sections of BDL or SHAM mice. Livers were double stained for PlGF (green) and CD31 (endothelial cells marker), F4/80 (macrophages), or α-SMA (myofibroblasts). Original magnification: 200×. **(D)** Western blotting analysis of PlGF expression in livers from mice at day of 14, 21, and 28 after BDL. The results normalized relative to the expression of β-actin. **(E)** Quantitative RT-PCR comparing relative levels of PlGF mRNA expression in livers from mice at day of 14, 21, and 28 after BDL or SHAM. Gene expression was normalized against GAPDH and folds were fold increase over SHAM (**P < 0.01; ***P < 0.001).

Male BALB/c mice (8–10 weeks) were purchased from Shanghai Laboratory Animal Research Center (Shanghai, China). The experimental protocol was performed in accordance with the guiding principles for the care and use of laboratory animals approved by the Fudan University Animal Care Committee and all animals received humane care. The animals were kept in an environmentally controlled room (23 ± 2°C, 55 ± 10% humidity) with a 12-h light/dark cycle and allowed free access to food and water. Mice were subjected to bile duct ligation (BDL) to induce liver fibrosis, while controls were sham-operated (SHAM) (27, 28). Mice were randomly distributed in four groups as shown in experimental design (Figure 1A). To deliver each siRNA, *in vivo* ready siRNAs were mixed with Invivofectamine 2.0 regents and injected in a volume of 100 µl at a dose of 5 mg/kg for three cycles starting 2 days after BDL surgery. Six to ten mice of each group were sacrificed on days 14, 21, and 28 after BDL, respectively; and the livers were removed and cut into small pieces and either snap-frozen in liquid nitrogen for storage at −80°C or fixed in freshly prepared 4% paraformaldehyde for 24 h at 4°C. Mouse sera were isolated to assay for liver functions.

### Cells Treatment

RAW 264.7 murine cells (Sigma, St. Louis, MO, USA) were grown in 150 cm^2^ flasks in DMEM supplemented with 10% FBS, 2 mM l-glutamine, 50 U/ml penicillin, and 50 µg/ml streptomycin. All incubations were performed in cells under the three or four passages. In experiments to assess the effects of PlGF on cells function, cells were transferred to 6-well plates at a density of 2.5 × 10^5^ cells/well in serum-free medium under a humidified 5% CO_2_ atmosphere at 37°C for 24 h. Then cells were washed and incubated with PBS vehicle, LPS (100 ng/ml), and recombinant mouse PlGF (rPlGF) at 50 ng/ml for 24 h at 37°C, respectively. To block the PlGF/VEGFR1 signaling, neutralizing antibody against mouse VEGFR1 (R&D Systems Inc., Minneapolis, MN, USA; 10 µg/ml) was applied where indicates. The cells were harvested for immunofluorescence analysis, RNA harvesting, and protein isolation. Staining and quantitative RT-PCR analysis were performed on three independent experiments. All measurements were performed in triplicate wells.

### Cell Migration Assay

To test migration, cells were investigated using a modified Boyden chamber assay (23, 28). Briefly, RAW 264.7 cells (5 × 10^4^ cells/well) were added to the upper chamber in DMEM without serum and exposed to rPlGF (25, 50, and 100 ng/ml) or LPS or PBS vehicle in the lower chamber. After 24 h of incubation at 37°C, cells on the upper membrane surface were removed and migratory cells on the membrane underside were fixed using 4% paraformaldehyde and stained using Crystal Violet Staining Solution (Beyotime Institute of Biotechnology, Nantong, China). Filter inserts were inverted and the number of migratory cells on the membrane underside was counted manually. The cells’ migration ability was expressed as the average cell number in eight randomly chosen fields at 200× (Olympus BX45, Olympus Corporation, Tokyo, Japan).

### Liver Enzymes Assays and Hydroxyproline Concentration

Serum alanine aminotransferase (ALT) and aspartate aminotransferase (AST) concentrations were determined spectrophotometrically using an automatic biochemical analyzer (Beckman, Fullerton, CA, USA). Hydroxyproline was measured in liver tissue hydrolyzates using the Hydroxyproline Assay Kit (BioVision, CA, USA) according to the manufacturer’s instructions; and the results are expressed as a microgram of hydroxyproline per gram of liver tissue.

### Histopathologic Evaluation, Immunohistochemistry (IHC)

In all experiments, the left liver lobe was excised and fixed with 10% neutral-buffered formalin, embedded in paraffin, and cut into 5-µm thick sections for histological analysis or IHC. Liver sections were stained with H&E and Sirius red according to standard procedures. Portal inflammation was graded with a 0–3 scale as described previously (22, 27). Fibrosis was quantified using ImageJ software 1.49 (NIH, Bethesda, MD, USA) on 10 non-contiguous Sirius red-stained sections and by the Scheuer modified histological activity index scoring system (5, 22, 27). A liver pathologist without knowledge of the treatment group examined histology. Protocol for IHC is described in detail in the supplementary experimental procedures (Figure S1 in Supplementary Material).

### Immunofluorescence Staining

The dissected liver tissues retrieved were fixed in 4% paraformaldehyde solution for 30 min, washed with PBS (pH 7.4), embedded in optimum cutting temperature tissue compound (OCT compound, Sakura, Japan), and frozen at −80°C for 1 day. Then the sections (10 µm in thickness) were cut with a cryotome Cryostat (Leica, CM 1900, Germany) and placed on slides for immunofluorescence staining. Blocking was performed in PBS with 3% BSA. The slides were incubated with antibody von Willebrandfactor (vWF) (Dako North American, Inc., Carpentaria, CA, USA), F4/80, α-SMA, or CD31 (Abcam, Cambridge, CA, USA) at the dilution of 1:100 overnight at 4°C and, subsequently, incubated with antibody PlGF or VEGFR1 (Abcam, Cambridge, CA, USA) at the dilution of 1:200 for 1 h at room temperature (RT) in case of double-staining. Alexa Fluor 594 donkey anti-mouse and Alexa Fluor 488 Donkey anti-Rabbit secondary antibodies (Yeasen Biotech, Shanghai, China) were incubated at 1:200 in PBS for 1 h at RT. After washing with Tris-buffered saline for three times, the cell nuclei were counterstained with Dapi-Fluoromount-G™ (SouthernBiotech, Birmingham, AL, USA). Finally, the stained tissues were analyzed by fluorescence microscopy (BX51, Olympus, Japan).

RAW 264.7 cells plated on 24-well plates and cultured on cover glass slips were fixed and permeabilized for 10 min in 4% paraformaldehyde, 0.2% TritonX-100 in PBS. Non-specific binding was blocked with 3% BSA for 1 h at RT, and then the cells were incubated with primary antibodies for F4/80 (dilution 1:200) and VEGFR1 (dilution 1:100) overnight at 4°C. After washing twice in PBS, the cells were incubated with fluorescein-labeled secondary antibody for 1 h at RT in the dark. The nuclei were stained with DAPI in the dark for 40 min at RT. The slides were washed twice with PBS, covered with DABCO (Sigma-Aldrich, St. Louis, MO, USA), and imaged by fluorescence microscopy (IX51, Olympus, Japan).

### Quantitative Analysis of Histological Markers and Angiogenesis

The number of α-SMA-, Desmin-positive cells, and the intensity of collagen III immunostaining in tissue sections were quantified using five random non-overlapping fields (100×) of each slide and determined for six animals in each group, and the area of staining was calculated as a percentage of the total area using the software NIH ImageJ 1.49 as described previously (5, 22).

For quantification of the numbers of hepatic macrophages in sections, six non-overlapping randomly selected fields of view per slide at 400× magnifications (F4/80^+^ cells) or 200× magnifications (CD68^+^ and Ly6C^+^ cells) were examined and expressed as cells per fields of view; and five mice of each group were examined (22, 29, 30).

Microvascular density in the liver tissue was assessed by determining the count of CD31-labeled ECs in five areas from each liver section at 200× magnification and is expressed as the number of CD31-positive vessels per field (22, 30). The vWF-positive cells were quantified using NIH ImageJ software; and five non-overlapping randomly selected fields of view per slide at 200× magnifications and eight mice of each group were examined (29, 30).

### Western Blot Analysis

Liver samples were homogenized in RIPA lysis buffer by adding protease inhibitor Cocktail (Roche) and phosphatase inhibitors Cocktail (Sigma), and then centrifuged at 10,000 *g* at 4°C for 20 min. Protein extraction from macrophage cells was as previously described (22). The protein concentration was measured using the Bicinchoninic Acid Protein Colorimetric Assay kits (BMI, Shanghai, China) with BSA as standard. Equivalent aliquots of protein samples (40 µg) were separated by electrophoresis on 7.5–12% SDS-PAGE gels and transferred onto polyvinylidenedifluoride membranes. The membrane was then incubated in blocking buffer (5% non-fat milk powder in TBST) for 3 h followed by incubation with primary antibody in TBST overnight at 4°C with the specific primary antibodies against PlGF, α-SMA, VEGFR1, CD31, TNF-α, IL-1β, TLR4, TLR9, HIF-1α, MCP-1, VCAM-1, ICAM-1 (all from Abcam, Cambridge, CA, USA), and CXCL10 (R&D Systems Inc., Minneapolis, MN, USA) at 1:1,000 dilution. The membrane was washed with TBST and then incubated with goat anti-rabbit, anti-mouse, or anti-rat secondary antibodies (Biotech Well, Shanghai, China; 1:1,500 dilution) for 2 h at RT. GAPDH or β-actin (Cell Signaling Technology, Boston, MA, USA; 1:5,000 dilution) was used as internal control, respectively. After washing off the unbound antibody with TBST, the expression of the antibody-linked protein was determined by an ECL™ Western Blotting Detection Reagents (Amersham Pharmacia Biotech Inc., Piscataway, NJ, USA). The densitometric analysis was performed with ImageJ.

### RNA Extraction and Quantitative RT-PCR

Total RNA was extracted from frozen liver tissues (caudate lobe) and cultured cells using Trizol reagent (Life Technologies, Grand Island, NY, USA) following manufacturer’s protocol. RNA was extracted reverse-transcribed with random hexamers and avian myeloblastosis virus reverse transcriptase using a commercial kit (Perfect Real Time, SYBR^®^ PrimeScriP™TaKaRa, Japan). Quantitative RT-PCR was performed for assessment of mRNA expression on an ABI Prism 7500 Sequence Detection system (Applied Biosystems, Tokyo, Japan) according to the manufacturer’s protocol. Probes and primers for target genes were purchased from Sangon Biotech Co., Ltd. (Shanghai, China, Table S1 in Supplementary Material). SYBR green gene expression assays were used to quantify target genes. The relative changes normalized to GAPDH mRNA using the formula 2^−ΔΔCt^, where ΔΔCt represents ΔCt values normalized with the mean ΔCt of control samples.

### Statistical Analysis

Data are expressed as mean ± SD. Statistical analyses were performed by using Graphpad Prism7 software (La Jolla, CA, USA). Comparisons between two independent groups were performed using a two-sample *t*-test. Comparisons between multiple groups were performed by one-way analysis of variance with *post hoc* Tukey’s multiple comparison tests or by two-tailed unpaired Student’s *t*-tests. A *P* value less than 0.05 was considered statistical significance.

## Results

### PlGF Is Highly Induced in Fibrotic Liver and PlGF Silencing Robustly Limits Intrahepatic PlGF Overexpression in BDL Mice

To examine the role of PlGF in chronic liver injury and fibrosis, we have used a well-established animal model of liver fibrosis induced by BDL (Figure 1A). As shown in Figure 1B, IHC staining revealed that PlGF expression was undetectable in SHAM mice and dramatically increased in non-parenchymal cells of the fibrotic liver as fibrosis progression and minimally in hepatocytes, particularly remarkable at the portal tracts and fibrous septa at 28 days of BDL. Notably, in livers of BDL mice, PlGF immunofluorescence co-localized with α-SMA and in cells located hepatic sinusoids, suggesting that PlGF expression is upregulated in profibrotic myofibroblasts; and we also noted that PlGF slightly expressed in macrophages (F4/80^+^) and in hepatic sinusoidal EC in sinusoids (Figure 1C).

To identify the role of PlGF in liver inflammation and fibrosis, we silenced PlGF *in vivo* using a chemically synthesized short, double-stranded RNA, which having well-defined structure with a phosphorylated 5′ end and hydroxylated 3′ ends with two overhanging siRNA to target hepatic PlGF expression. After 2 days of BDL, mice were injected with PlGF-specific or NTC siRNA by Invivofectamine reagent (Figure 1A). Efficiency of knockdown of PlGF *in vivo* by using siRNA was assessed by IHC and Western blotting; and the IHC staining signal of PlGF was obviously weak in fibrotic livers from PlGF siRNA-treated mice when compared with the livers from control and NTC siRNA-treated mice (Figure 1B). Consistent with our histological finding, Western blot results confirmed that targeted siRNA treatment resulted in a significant decrease in intrahepatic PlGF expression at different stages of disease progression (days 14, 21, and 28 after BDL) (Figure 1D). Moreover, to further ascertain the effect of siRNA-mediated suppression of PlGF expression *in vivo*, we also analyzed liver PlGF mRNA levels at 14, 21, and 28 days after BDL. Our results demonstrated that the levels of PlGF mRNA expression were gradually increased following BDL; which were significantly downregulated at their corresponding time points by PlGF siRNA administration (Figure 1E).

### PlGF Silencing Reduces Liver Injury, Inflammation, and Fibrosis in BDL Mice

**Figure 2:**
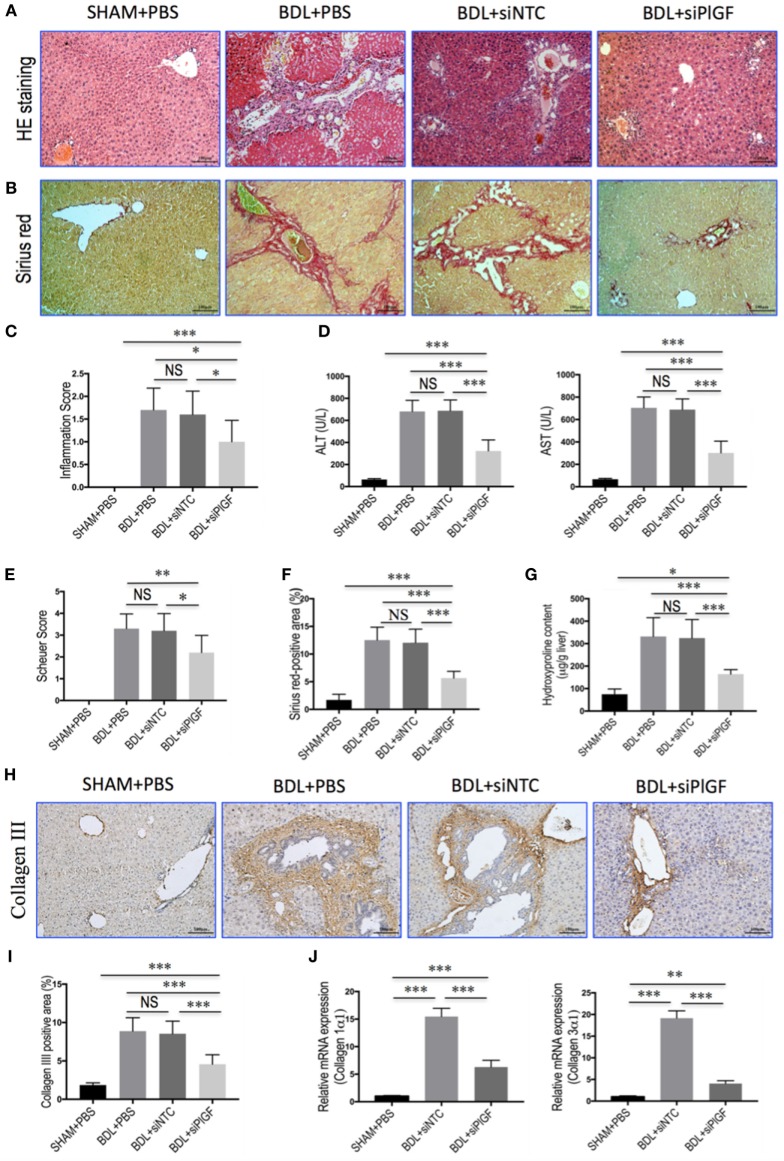
Placental growth factor (PlGF) silencing reduces liver injury, inflammation, and fibrosis in bile duct ligation (BDL) mice. **(A)** H&E stained in liver sections (original magnification: 100×). Mice underwent SHAM or BDL surgery and received small interfering RNA or PBS and sacrifice at 28 days. **(B)** Sirius red staining of liver sections (original magnification: 100×). **(C)** Inflammation scores (*n* = 10/group). **(D)** Serum alanine aminotransferase (ALT) and aspartate aminotransferase (AST) concentrations in mice from each group (*n* = 8–10/group). **(E)** Assessment of liver fibrosis based on Scheuer’s scoring system. **(F)** Hepatic fibrotic area based on Sirius red staining. **(G)** Liver hydroxyproline concentration (*n* = 8/group). **(H)** Representative microscopy images of collagen III immunohistochemistry (original magnification: 100×). **(I)** Quantitative analysis of collagen III positive area (*n* = 8/group). **(J)** Hepatic expression of collagen 1α1 and collagen 3α1 mRNA was determined by quantitative RT-PCR. Results were normalized relative to GAPDH expression and expressed as mean ± SD fold increase over SHAM control mice. *P < 0.05; **P < 0.01; ***P < 0.001; NS, not significant.

Morphological analysis by H&E staining of liver sections from BDL mice revealed distortion of the normal architecture, with a marked aggregation of lymphocytes, severe hepatocytes necrosis, and proliferation of bile ductules. Mice presented with remarkable fibrosis (stage 3 or 4) showing the characteristic pattern of extensive portal–portal and portal–central fibrosis linkage following 4 weeks of BDL; whereas the SHAM mice shown normal architecture (Figures 2A,B). Impressively, however, PlGF silencing in BDL mice exhibited thinner septa, mild liver fibrosis, and more preserved hepatic parenchyma (Figures 2A,B). Moreover, in BDL mice, PlGF silencing decreased the severity of hepatic inflammation compared with those of NTC siRNA-treated group (1.60 ± 0.52 vs. 1.00 ± 0.47, respectively, *P* = 0.021; Figure 2C). This was indeed also supported by the findings that serum ALT and AST levels were decreased in BDL mice receiving PlGF siRNA (Figure 2D).

As revealed by the histological analysis of liver sections, there was a lower mean fibrosis score in BDL mice receiving PlGF siRNA treatment compared with those of the mice receiving NTC siRNA treatment (2.2 ± 0.8 vs. 3.2 ± 0.8, *P* = 0.011; Figure 2E). This was further confirmed by Sirius red-stained area analysis and hepatic hydroxyproline content, showing that PlGF silencing to BDL mice resulted in a 53.2% reduction in Sirius red-stained area (5.64 ± 1.21% vs. 12.05 ± 2.44%, *P* < 0.0001; Figure 2F) and a 49.5% reduction in hepatic hydroxyproline content (Figure 2G) compared to those animals receiving NTC siRNA. In addition, IHC evaluation showed that the deposition of collagen III was increased in the portal tracts, septa, and perisinusoidal spaces of the lobules in BDL mice, whereas PlGF siRNA treatment attenuated collagen III accumulation in livers (Figure 2H). These findings were supported by quantification of collagen III positive areas showing a decrease in the areas by 46.5% in fibrotic liver sections from PlGF siRNA-treated mice compared those from NTC siRNA-treated animals (Figure 2I). We also examined the gene expression of collagen 1α1 and collagen 3α1, suggesting the levels of both genes were significantly downregulated by siRNA-mediated PlGF knockdown in BDL mice (Figure 2J).

Taken together, these results suggested that PlGF silencing led to a significant reduction in BDL-induced liver inflammation and fibrogenesis in mice.

### PlGF Silencing Inhibits Activation of HSCs in BDL Mice

**Figure 3:**
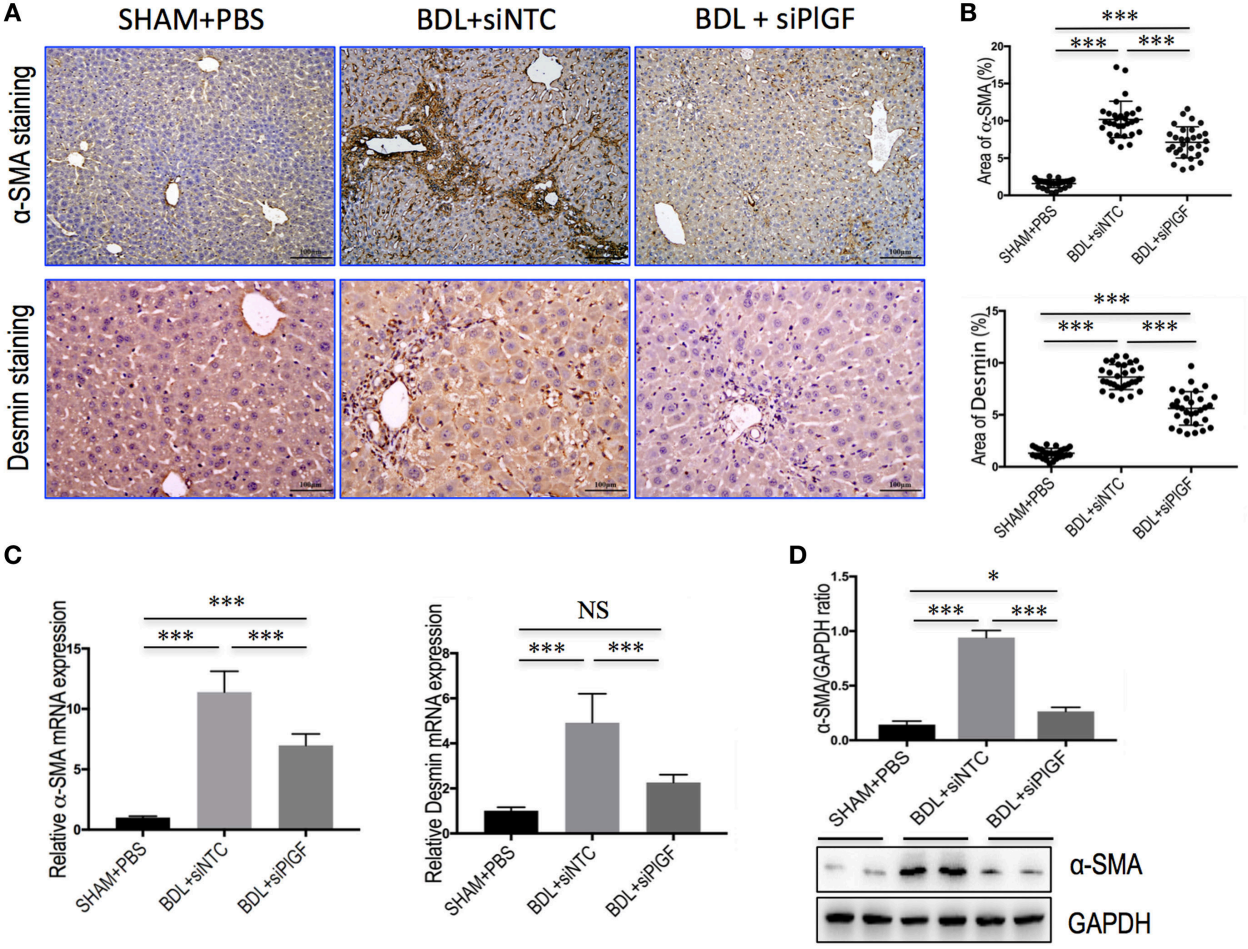
Placental growth factor (PlGF) silencing inhibits activation of hepatic stellate cells in bile duct ligation (BDL) mice. **(A)** Representative microscopy images of α-SMA staining (original magnification: 100×) and Desmin staining (original magnification: 200×) in livers. Mice were induced liver fibrosis by BDL for 28 days and treated with small interfering RNA or PBS. **(B)** Quantification of α-SMA- and Desmin-positive area by ImageJ software (NIH). Results mean of six fields and *n* = 5/group. **(C)** Hepatic α-SMA and Desmin mRNA expression was determined by quantitative RT-PCR (*n* = 5). Results were normalized relative to GAPDH expression and expressed as mean ± SD fold change over SHAM control mice. **(D)** Western blot analysis of hepatic α-SMA expression and GAPDH as loading control. *P < 0.05; ***P < 0.001; NS, not significant.

Activated HSCs are considered central ECM-producing cells within the injured liver and also involved in the pathologic angiogenesis and vascular remodeling (22, 23). We found that PlGF silencing induced a significantly reduction in BDL-induced expression of the marker of activated HSCs, α-SMA, and Desmin as shown by IHC analysis (Figure 3A). Moreover, computer-assisted semiquantitative analysis demonstrated that the number of α-SMA- and Desmin-positive cells was significantly lower in livers from PlGF siRNA-treated BDL mice than those from NTC siRNA-treated BDL mice (Figure 3B). These findings were substantiated by quantitative RT-PCR experiments, suggesting the levels of α-SMA and Desmin mRNA transcript in fibrotic livers were correspondingly reduced following PlGF knockdown (Figure 3C). In addition, we also examined α-SMA protein expression by western blotting, indicating PlGF silencing with siRNA inhibited the α-SMA protein expression *in vivo* (Figure 3D). Collectively, these *in vivo* data indicated that PlGF silencing efficiently inhibited myofibroblastic activation of HSC during BDL-induced liver injury and fibrosis.

### PlGF Silencing Attenuates Hepatic Angiogenesis in BDL Mice

**Figure 4:**
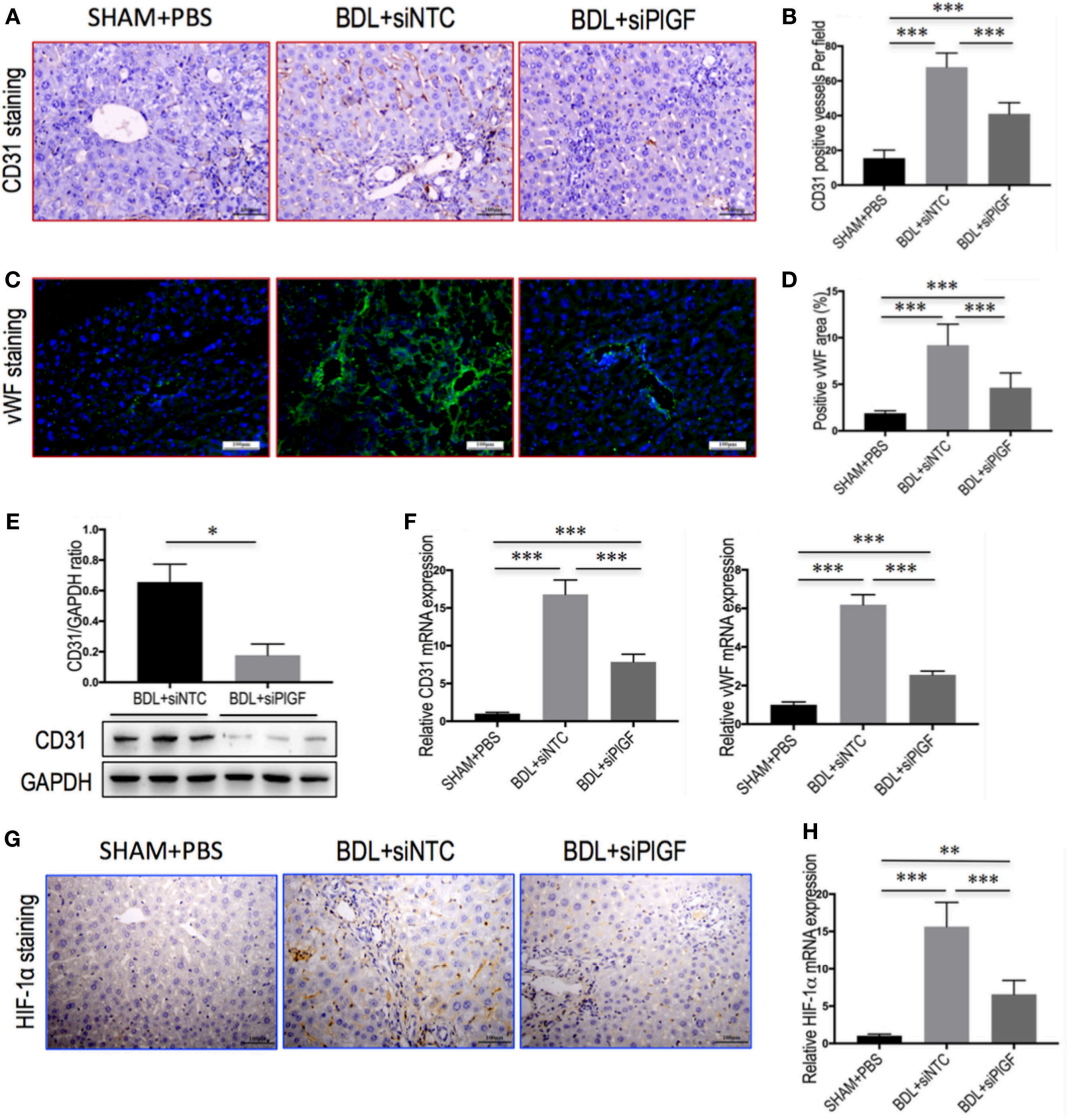
Placental growth factor (PlGF) silencing attenuates hepatic angiogenesis in bile duct ligation (BDL) mice. Mice were induced liver fibrosis by BDL for 28 days and treated with small interfering RNA or PBS. **(A)** Representative images of intrahepatic CD31 immunohistochemistry (IHC) staining in mice from each group (original magnification: 200×). **(B)** Microvessel density was assessed by counting CD31-positive vessels (*n* = 8/group). **(C)** Representative images of intrahepatic von Willebrandfactor (vWF) immunofluorescence staining in mice from each group (original magnification: 200×). **(D)** Microvessel density was assessed by vWF-positive vessels area in livers (*n* = 8/group). **(E)** Western blot analysis of hepatic CD31 expression and GAPDH as loading control (*n* = 3). **(F)** Hepatic CD31 and vWF mRNA expression were determined by quantitative RT-PCR. Results were normalized relative to GAPDH expression and expressed as mean ± SD fold change over SHAM control mice (*n* = 5). **(G)** Representative images of intrahepatic HIF-1α IHC staining in mice from each group (original magnification: 200×). **(H)** Hepatic HIF-1α mRNA expression was determined by quantitative RT-PCR. Results were normalized relative to GAPDH expression and expressed as mean ± SD fold change over SHAM control mice (*n* = 5) (*P < 0.05; **P < 0.01; ***P < 0.001).

To investigate the vascular changes in PlGF silencing, we conducted studies to determine hepatic neovascularization and the expression of angiogenic factors in livers. The EC marker CD31 was expressed in the endothelium of the veins and in the central veins in livers of sham-operated mice, but not along the sinusoids; and challenge mice by BDL at 28 days led to a markedly increased number of CD31-positive vessels in livers (Figure 4A). However, CD31-positive EC staining in BDL siPlGF liver sections was significantly less than in the BDL siNTC group as evidenced in mean microvessels density (67.9 ± 8.1 vs. 41.1 ± 6.4/per field, *P* < 0.001) (Figure 4B). These results were further supported by the levels of CD31 mRNA and protein expression in livers; showing that hepatic angiogenesis was inhibited by PlGF silencing in fibrotic mice (Figures 4E,F). Similar histologic pattern was observed in vWF staining of tissues, indicating upregulated in livers from BDL mice; however, BDL mice receiving PlGF-siRNA exhibited decrease in the intensity of vWF staining and vWF-positive vessels area in livers (Figures 4C,D), consistent with the decrease in vWF gene expression (Figure 4F). In addition, we noted a significant decrease in expression of HIF-1α in fibrotic animals treated with PlGF siRNA as shown by IHC (Figure 4G) and Western blot analysis for HIF-1α confirmed the morphological changes observed (Figure S1 in Supplementary Material). Similarly, hepatic HIF-1α mRNA levels in BDL mice were also significantly reduced by PlGF silencing with siRNA *in vivo* (Figure 4H). Together, these results indicated that PlGF silencing effectively attenuates pathologic vascular changes that occur in response to BDL.

### PlGF Silencing Reduces Hepatic Macrophage Recruitment in BDL Mice

**Figure 5:**
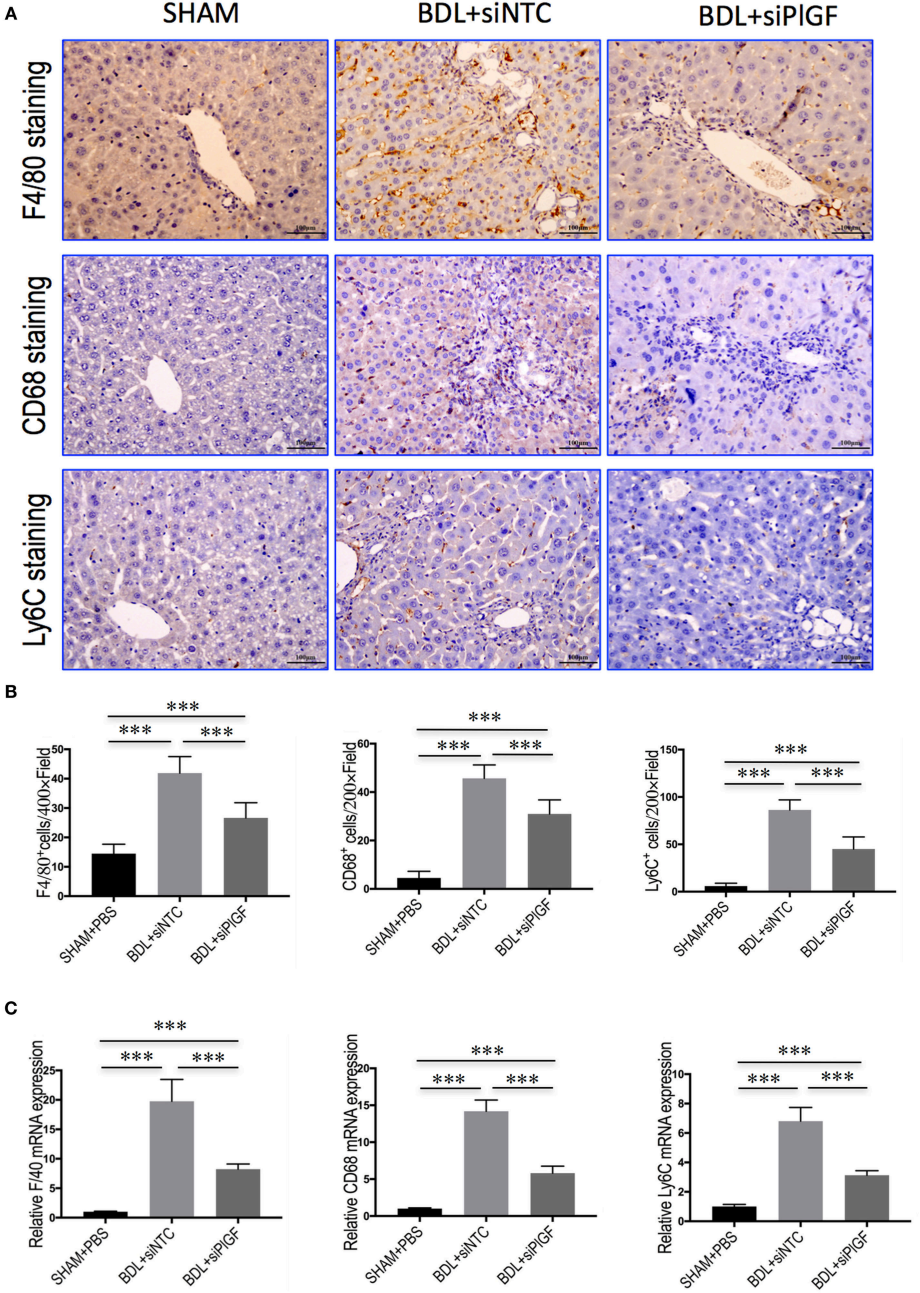
Placental growth factor (PlGF) silencing reduces hepatic macrophage recruitment to the liver in bile duct ligation (BDL) mice. **(A)** Immunohistochemical detection of F4/80-, CD68-, and Ly6C-positive cells in liver sections (original magnification: 200×). Mice were induced liver fibrosis by BDL for 28 days and treated with small interfering RNA or PBS. **(B)** Quantization of F4/80-, CD68-, and Ly6C-positive cells in liver sections. Results mean of six fields and *n* = 5/group. **(C)** Hepatic expression of F4/80, CD68, and Ly6C mRNA was determined by quantitative RT-PCR, and the results are shown as fold change compared with sham-operated (SHAM) control and GAPDH served as loading control (*n* = 5) (***P < 0.001).

To investigate the mechanism of PlGF silencing in the pathogenesis of hepatic inflammation and fibrosis-associated angiogenesis, we explored the markers of monocytes and macrophages infiltration in fibrotic livers. Compared to the SHAM mice, IHC staining for the macrophage markers indeed revealed BDL-enhanced infiltration of F4/80^+^ or CD68^+^ macrophages into fibrotic livers. Remarkably, however, the increase of hepatic macrophages infiltration was significantly reduced by PlGF siRNA treatment in BDL mice when compared with those by NTC siRNA treatment mice (Figure 5A); and the results were further confirmed by quantification of the F4/80^+^ or CD68^+^ staining cells (Figure 5B). Moreover, these results consistent with the genes expression of F4/80 and CD68, demonstrating PlGF silencing in BDL mice strikingly decreased the upregulated F4/80 and CD68 mRNA levels (Figure 5C). In addition, the total number of Ly6C^+^ cells, a marker for BM-derived circulating peripheral blood monocytes, was also significantly higher in fibrotic livers in BDL mice than normal livers from the SHAM mice. However, PlGF silencing led to reduced Ly6C^+^ macrophage infiltration (Figures 5A,B). Similarly, hepatic Ly6C mRNA expression in BDL mice was also inhibited by PlGF silencing with siRNA *in vivo* (Figure 5C). Taken together, these results suggested that siRNA-mediated PlGF knockdown significantly reduced hepatic macrophage recruitment to the livers, which being responsible for attenuating liver inflammation and fibrosis.

### PlGF Silencing Inhibits Macrophages Activation and Inflammatory Properties in BDL Mice

**Figure 6:**
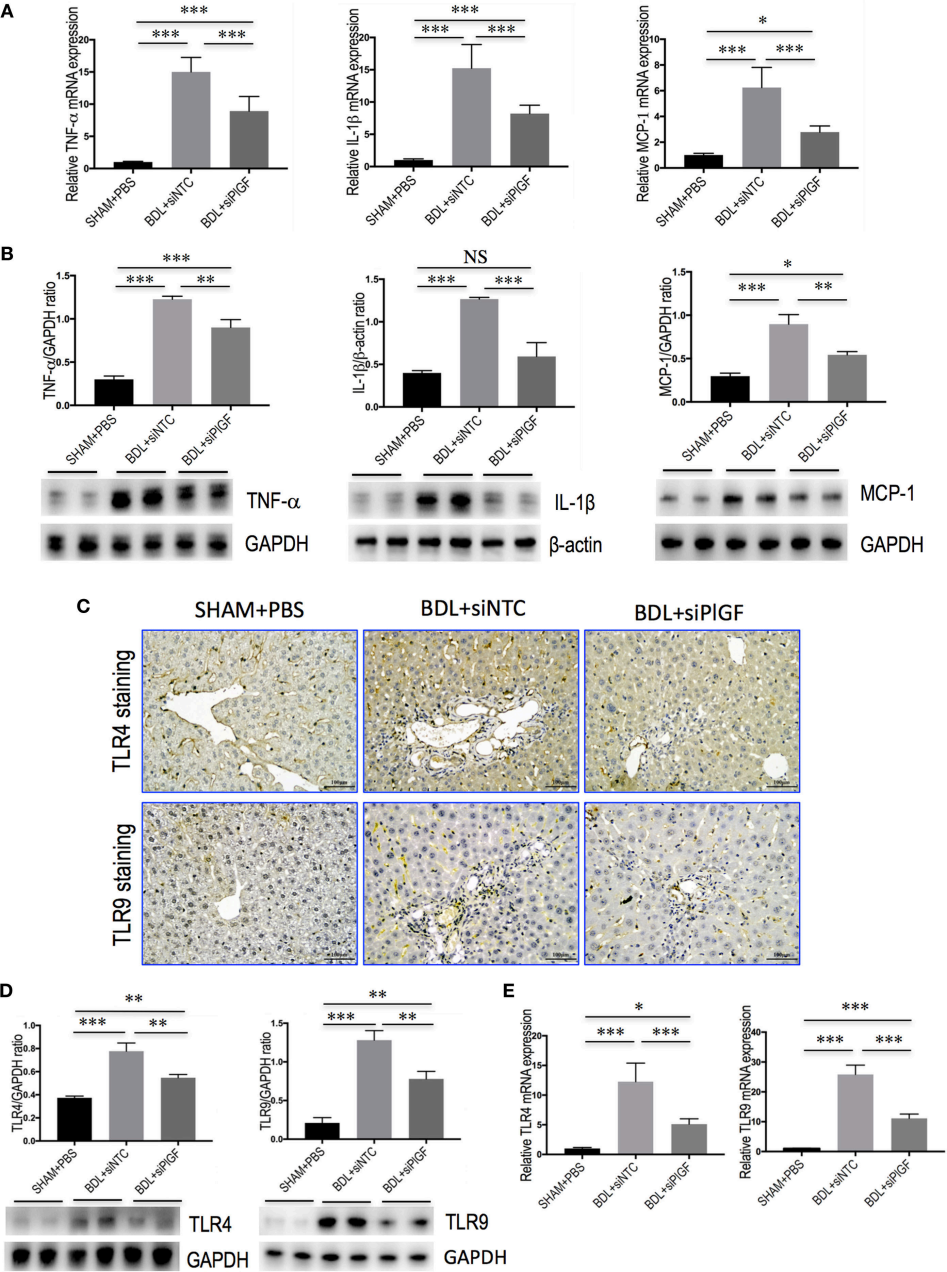
Placental growth factor (PlGF) silencing reduces hepatic macrophages activation and inflammatory properties in bile duct ligation (BDL) mice. Mice were induced liver fibrosis by BDL for 28 days and treated with small interfering RNA or PBS. **(A)** Hepatic expression of TNF-α, IL-1β, and MCP-1 mRNA was determined by quantitative RT-PCR, and results are shown as fold change compared with sham-operated (SHAM) control and GAPDH served as loading control (*n* = 5). **(B)** Western blot analysis of hepatic TNF-α, IL-1β, and MCP-1 protein expression, with results normalized relative to the expression of GAPDH or β-actin (*n* = 3). **(C)** Immunohistochemical staining for TLR4 and TLR9 in livers (original magnification: 200×). **(D)** Western blotting analysis of hepatic TLR4 and TLR9 protein expression with results normalized relative to the expression of GAPDH (*n* = 3). **(E)** Hepatic expression of TLR4 and TLR9 mRNA was determined by quantitative RT-PCR, and the results are shown as fold change compared with SHAM control and GAPDH served as loading control (*n* = 5). *P < 0.05; **P < 0.01; ***P < 0.001; NS, not significant.

To investigate whether PlGF mediates liver inflammation through switching macrophages subtypes and regulating their function, we examined the expression of pro-inflammatory cytokines associated with M1 macrophages in the liver of fibrotic mice, such as TNF-α, IL-1β, and MCP-1. Our results demonstrated that hepatic expression of TNF-α, IL-1β, and MCP-1 mRNA was strongly upregulated in BDL fibrosis models. However, BDL mice receiving siPlGF reduced TNF-α, IL-1β, and MCP-1 mRNA by 6.1-fold, 7.0-fold, and 3.5-fold, respectively (Figure 6A). Those findings were supported by our Western blot analysis, demonstrating that the increase of these chemokines in fibrotic liver was indeed attenuated by PlGF silencing (Figure 6B).

In addition, we also examined the expression of TLR4 and TLR9 in livers. IHC data showed that weak constitutive expressions of TLR4 or TLR9 on sinusoidal ECs of SHAM mice livers, with hepatocytes showing no or only slight expression (Figure 6C). After 4 weeks BDL, increased TLR4 or TLR9 expression in livers was markedly observed in the periportal and interlobular septa, as well as increased expression on interstitial space between hepatocytes. However, giving PlGF siRNA to BDL mice resulted in moderate staining for TLR4 and TLR9 (Figure 6C). Consistent with these results, the expression of TLR4 and TLR9 protein was obviously upregulated in livers of BDL mice; however, siPlGF treatment to BDL mice decreased hepatic TLR4 and TLR9 expression when compared with vehicle treatment (Figure 6D). Similar results were seen in TLR4 and TLR9 mRNA expression, indicating PlGF silencing markedly reduced both gene expression (Figure 6E).

**Figure 7:**
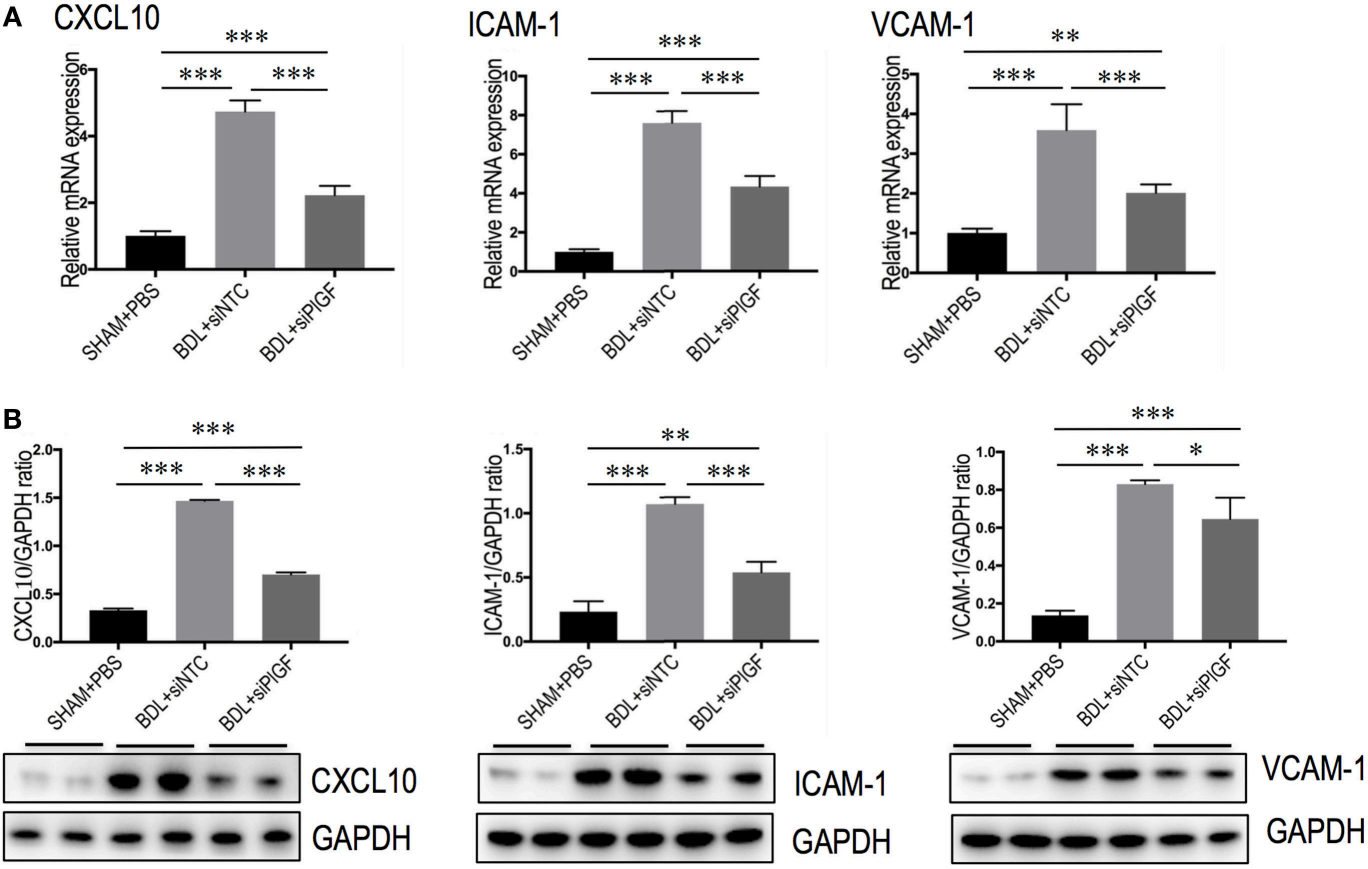
Placental growth factor (PlGF) knockdown attenuates the expression of pro-inflammatory adhesive molecules in the vasculature of fibrotic mice. **(A)** Hepatic expression of CXCL10, intercellular adhesion molecule 1 (ICAM-1), vascular cell adhesion molecular-1 (VCAM-1) mRNA expression was determined by quantitative RT-PCR, and results are shown as fold change compared with sham-operated (SHAM) control. GAPDH served as loading control (*n* = 5). **(B)** Western blotting analysis of CXCL10, ICAM-1, and VCAM-1 protein expression in lysed liver tissue from each group mice, with results normalized relative to the expression of GAPDH (*n* = 3) (*P < 0.05; **P < 0.01; ***P < 0.001).

To further understand the link between PlGF knockdown and the reduction in inflammatory infiltrate, the expression of pro-inflammatory adhesive molecules, such as CXCL10, ICAM-1, and VCAM-1 in the vasculature of fibrotic mice was also analyzed. We found that the levels of CXCL10, VCAM-1, and ICAM-1 mRNA expression in livers were markedly enhanced in BDL mice received siNTC compared with SHAM mice, but these increase in livers were attenuated by PlGF siRNA treatment to BDL mice (Figure 7A). Meanwhile, those finding were supported by our western blotting, demonstrating that the increase of these chemokines in BDL-induced fibrotic livers was indeed attenuated by PlGF silencing (Figure 7B).

Taken together, these results suggest that PlGF might play a key role in the activation of Kuffer cells/macrophages in liver upon chronic injury and substantially produce a variety of pro-inflammatory cytokines and chemokines.

### PlGF Promotes Hepatic Macrophage Recruitment and Activation *via* VEGFR1

**Figure 8:**
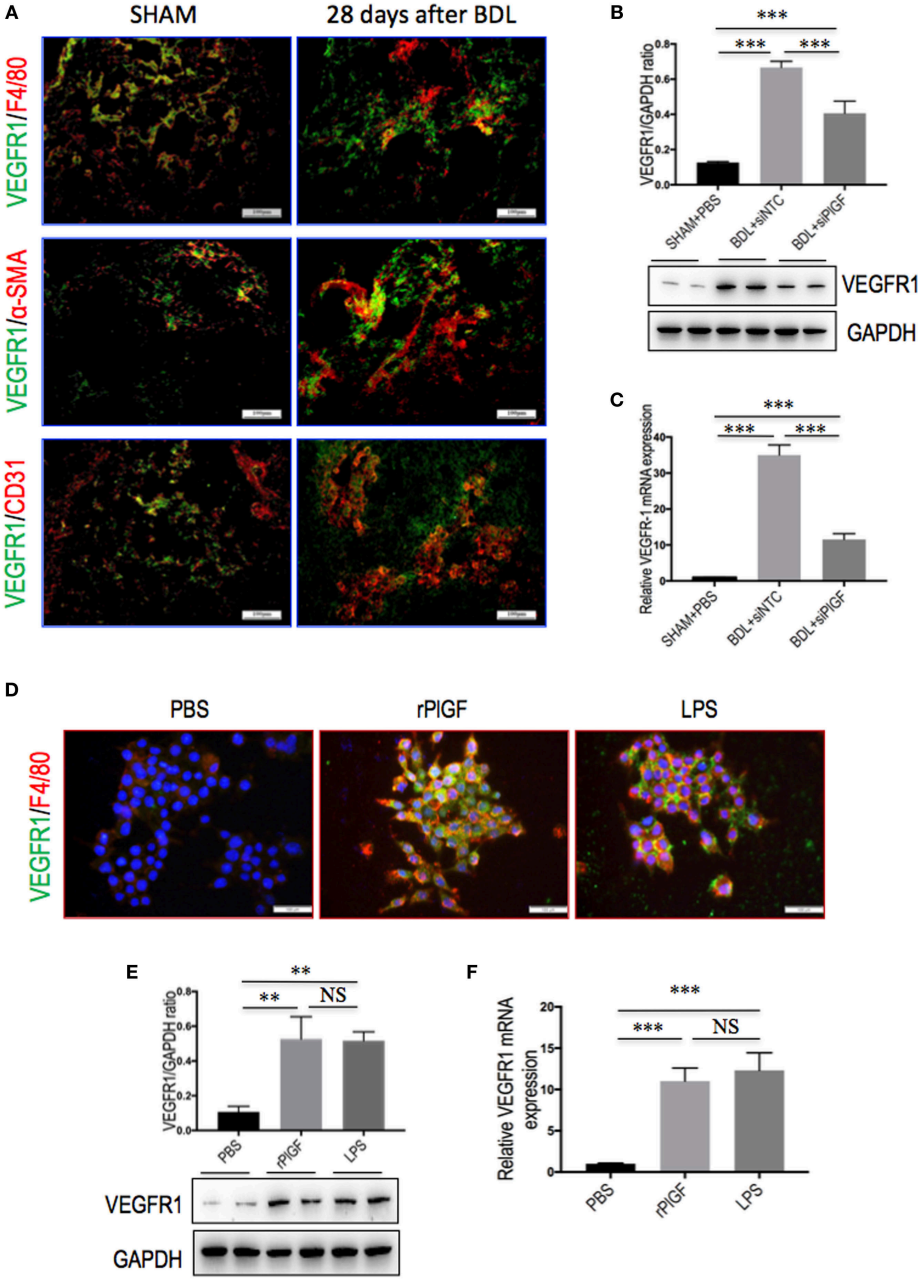
The expression and distribution of VEGR1 in fibrotic livers and in macrophages. **(A)** Immunofluorescent double staining of vascular endothelial growth factor receptor 1 (VEGFR1) in liver sections of bile duct ligation (BDL) or sham-operated (SHAM) mice at 28 days. Livers were double stained for VEGFR1 (green) and CD31 (endothelial cells marker), F4/80 (macrophages), or α-SMA (myofibroblasts). Original magnification: 200×. **(B)** Western blotting analysis of VEGFR1 expression in lysed liver tissues, with results normalized relative to the expression of GAPDH (*n* = 3). **(C)** Hepatic VEGFR1 mRNA expression was measured by quantitative RT-PCR. Results are shown as fold change compared with SHAM control and GAPDH served as loading control (*n* = 5). **(D)** Double immunofluorescent expression of VEGFR1 (green) and macrophages marker F4/80 (red) in RAW 264.7 cell line. Cells were stimulated with rPlGF (50 ng/ml) or lipopolysaccharide (LPS) (100 ng/ml) for 24 h, respectively. DAPI as blue nuclear counterstain. Scale bar = 100 µm for each picture. **(E)** Western blot analysis for VEGFR1 in macrophages RAW 264.7 cells stimulated with rPlGF (50 ng/ml) or LPS (100 ng/ml) for 24 h. PBS and LPS serve as negative and positive controls, respectively. **(F)** The levels of VEGFR1 mRNA RT-PCR expression in RAW 264.7 cell were measured by quantitative RT-PCR. Cells stimulated with rPlGF (50 ng/ml) or LPS (100 ng/ml) for 24 h. The mRNA levels were normalized to GAPDH mRNA levels and presented as fold stimulation (mean ± SD) vs. PBS (**P < 0.01; ***P < 0.001; NS, not significant).

Placental growth factor exclusively binds to VEGFR1 and not VEGFR2 (17, 18), we, therefore, investigated whether VEGFR1 signaling in macrophages mediated the role of PlGF in liver inflammation and fibrosis *in vivo*. First, we investigated the cellular source of VEGFR1 in fibrotic livers; and our double staining of liver sections for VEGFR1 and CD31, F4/80, or α-SMA revealed obviously increased expression VEGFR1 in those non-parenchymal cells in mice of BDL, whereas there has weak expression in those cells in livers from SHAM mice (Figure 8A). Next, we further examined levels of VEGFR1 in fibrotic livers at 4 weeks of BDL mice by both quantitative RT-PCR and Western blot analysis, respectively. Our results showed that a marked increase in VEGFR1 mRNA and protein expression was demonstrated with the development of hepatic fibrosis in BDL mice compared with SHAM control (Figures 8B,C). However, PlGF silencing significantly downregulated the expression of VEGFR1 at gene levels and at protein levels in fibrotic livers when compared to NTC siRNA-treated fibrotic mice (Figures 8B,C).

**Figure 9:**
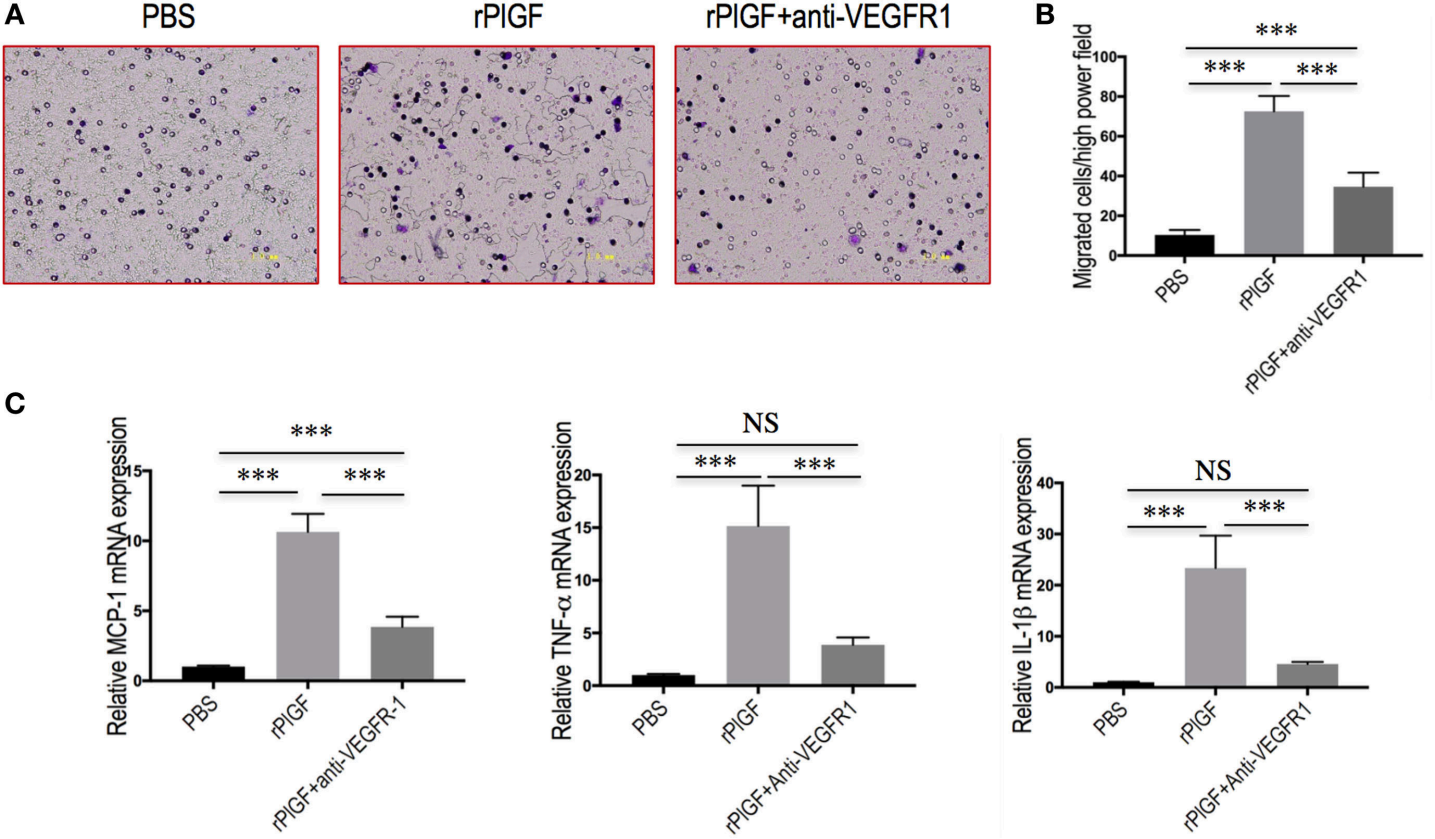
Placental growth factor (PlGF) promotes hepatic macrophage recruitment and polarization *via* vascular endothelial growth factor receptor 1 (VEGFR1) signaling. **(A)** Migration of macrophages toward PlGF was assessed in Boyden’s chamber experiments. Original magnification: 200×. **(B)** Migration of macrophages into the lower chamber was quantified 24 h after stimulation. Media only, PlGF (50 ng/ml) or PlGF + VEGFR1 blocking antibody (10 µg/ml) for 24 h. **(C)** The levels of MCP-1, TNF-1α, and IL-1β mRNA expression in RAW 264.7 cell were measured by quantitative RT-PCR. The mRNA levels were normalized to GAPDH mRNA levels and presented as fold stimulation (mean ± SD) vs. PBS. ***P < 0.001; NS, not significant.

To further confirm whether VEGFR1 expression on macrophages was involved in macrophages recruitment or activation upon liver injury, we tested the migratory response and inflammatory properties of mouse macrophages RAW 264.7 cell line using an *in vitro* transmigration assay with recombinant mouse PlGF (rPlGF). First, we evaluated the effect of PlGF on VEGFR1 expression from macrophages, we selected LPS as positive control since LPS is known to activate macrophages *in vitro*. Indeed, rPlGF stimulated VEGFR1 expression in RAW 264.7 cells as shown by double immunofluorescent staining (Figure 8D) and Western blotting (Figure 8E), consistent with increased expression of VGFR1 in livers from BDL mice. Similarly, the levels of VEGFR1 mRNA expression were also increased by rPlGF in a similar manner in LPS-treated (Figure 8F). Second, RAW 264.7 cells were treated for 24 h with rPlGF (50 ng/ml) and there was 6.98-fold increase in RAW 264.7 cell migration toward rPlGF in Boyden assays compared to cells migrating toward vehicle (Figures 9A,B). The 24-h incubation time and 50 ng/ml contents of PlGF were chosen on the basis of the results of a pilot studies (Figure S2 in Supplementary Material). Moreover, to determine whether PlGF could activate macrophages to generate cytokines, MCP-1, TNF-α, and IL-1β, we utilized an *in vitro* assay to mimic this situation. rPlGF treatment of RAW 264.7 cells for 24 h showed that the expression of MCP-1, TNF-α, and IL-1β mRNA were obviously increased as shown in our quantitative RT-PCR results (Figure 9C). Finally, to further confirm the role of PlGF in the migration and activation of macrophages, we blocked PlGF/VEGFR1 signaling axis with a specific VEGFR1 neutralizing antibody that was added to cultured cells. We found that the upregulation of inflammatory cytokines (MCP-1, TNF-α, and IL-1β) in macrophages at rPlGF challenge was reduced by VEGFR1 neutralizing antibody (Figure 9C), suggesting that PlGF/VEGFR1 signaling axis was strongly involved in activation of macrophages. Notably, the migratory capacity of macrophages was also significantly inhibited (52.2% reduction in mean cells number) while PlGF/VEGFR1 signaling was blocked (Figures 9A,B). Together, these results show clearly that PlGF promotes macrophage recruitment and activation upon liver injury *via* VEGFR1.

## Discussion

Currently, no effective therapy is available for liver fibrosis, and a better understanding of pathologic mechanisms regulating this disorder is urgently needed for identifying novel antifibrotic therapeutic agents (1–3). In this study, our findings lend support for the notion that PlGF plays a critical role in the pathogenesis of fibrotic liver disease and provide evidence that PlGF is a potential therapeutic target in chronic inflammatory liver diseases (22, 23). Moreover, knockdown of PlGF by siRNA in BDL mice ameliorates hepatic inflammation, angiogenesis, and fibrogenesis. Most importantly, these findings have provided new insights for understanding the mechanism of PlGF contributing to liver inflammation and fibrosis through promoting recruiting hepatic macrophage to the liver and enhancing inflammatory responses.

To date, it has been widely accepted that hepatic fibrosis develops as a response to chronic liver injury and almost exclusively occurs in a pro-inflammatory environment (1–4). Recent studies indicated that hepatic macrophages play important roles in the pathogenesis of hepatic inflammation and fibrosis (9–13, 29–32). During ongoing chronic injury and the progression of fibrosis, pro-inflammatory macrophages derived from monocytes prevail in the liver (12, 13, 32–34). Both monocyte-derived macrophages and Kupffer cells have profibrogenic properties, by promoting the activation and survival of HSCs and myofibroblasts through secreting both TGF-β and PDGF (33–35). Given that inflammatory macrophages can exacerbate chronic liver disease, a deeper understanding of the mechanisms by which macrophages promote inflammation and fibrosis might lead to novel strategies to treat liver diseases (12, 31, 33).

As is well-known that PlGF is a multitasking cytokine and is involved in BM-derived cell activation, endothelial stimulation, inflammation, pathologic angiogenesis, and wound healing (17–20, 36). We demonstrated that hepatic PlGF expression was remarkably increased in the BDL model (Figure 1); and PlGF and its main receptor VEGFR1 were upregulated in activated HSCs and macrophages (Figures 1 and 8); these results agree with the findings of others and our recent reports in CCl4 animal models (22, 23). Notably, our recent *in vitro* study demonstrated that hypoxia could induce PlGF overexpression dependent on HIF-1α during liver fibrosis, then promotes HSCs activation and proliferation through modulating PI3K/Akt signaling pathway (22). Therefore, it is likely that PlGF may also induce recruitment of monocytes and macrophages to the injury livers and promote macrophages activation, contributing to liver inflammation and HSCs activation during fibrosis development. As expected, our results indeed demonstrated that PlGF silencing suppressed the activation of HSCs (Figure 3) and reduced the severity of liver inflammation in BDL mice (Figure 2C), which leads to attenuate liver fibrosis and angiogenesis.

Besides PlGF directly amplifies HSC activation, in this study, we focused particularly on the role of PlGF in interactions between macrophages and HSC as well as in activation of HSC during fibrogenesis *in vivo*. Indeed, the results of this study indicated that PlGF silencing in BDL mice remarkably inhibited the activation of macrophages (Figures 6 and 7) or recruitment of CD68^+^, F4/80^+^, and Ly6C^+^ macrophages into the fibrotic liver (Figure 5), which were critically involved in the mechanism to explain the attenuated fibrosis and HSC activation observed in the treatment of PlGF silencing. Consistent with this notion, PlGF has been shown to promote monocyte infiltration in ischemic tissues, tumors, atherosclerotic plaques, and bone fractures (18, 37). Moreover, prior studies have demonstrated that inhibition of PlGF might also affect tumors by reducing TAM infiltration (18, 22–26). It also induces polarization of TAM to an M2-like proangiogenic phenotype, thereby promoting tumor vessel disorganization (17, 18, 38). In addition, the recruitment process is also mediated by other chemokines and its receptors, such as CXCL10, ICAM-1, VCAM, and CCR2 (33, 35, 39, 40). Although mice induced by BDL significantly increased mRNA and protein expression of CXCL10, ICAM-1, and VACM-1 in livers, this increase was obviously impacted by PlGF silencing (Figure 7). It is noteworthy that the CXC family of chemokines also operates in pathological angiogenesis preceding/perpetuating fibrosis (39, 40).

Liver injury triggers Kupffer cell activation, leading to inflammatory cytokines and chemokines release, which exert a key role in the process of liver fibrosis and angiogenesis (31, 35, 40). In line with lower levels of intrahepatic macrophages, pro-inflammatory cytokines, such as TNF-α, IL-1β, and MCP-1 were significantly reduced in liver tissue by knockdown of PlGF by siRNA in BDL mice (Figures 6A,B). Several independent studies highlighted the importance of the chemokines receptor CCR2 and its main ligand, MCP-1, for monocyte/macrophage recruitment during experimental hepatic fibrosis, suggesting that inhibition of CCR2 or MCP-1 might bear therapeutic potential in chronic liver diseases (8, 9, 33, 41). In addition, macrophages also express multiple toll-like receptors (TLRs)—such as TLR4 and TLR9, and it has been reported that TLRs interact with oxDNA and microbial components, such as LPS, Hsp60, and other ligands, and result in macrophage activation and the productions of pro-inflammatory mediators (such as TNF-α and MCP-1) (32, 35, 42–44). In this study, we also found that PlGF silencing inhibited the levels of TLR4 and TLR9 gene and protein expression in fibrotic liver after 4-week BDL (Figures 6C,E), contributing to amelioration of liver inflammation and fibrosis (42–44). Thus, these results indicated that the interaction of HSCs with pro-inflammatory cells such as Kupffer cells was a crucial event in HSCs activation and fibrosis (6–9, 29), while chemokines and their receptors were likely to serve as important contributors to this interaction (6–9, 25, 32, 44).

In addition, fibrosis is typically associated with impaired angiogenesis and sustained development of local tissue hypoxia (6, 22). Of note, hypoxia has been shown to be a profibrotic stimulus that contributes to the development of fibrosis and angiogenesis through an HIF-mediated pathway (6, 22), we also have demonstrated that HIF-1α was increased in fibrotic livers induced by BDL (Figure 4; Figure S1 in Supplementary Material); however, PlGF-specific siRNA inhibited the expression of HIF-1α in fibrotic livers, thus contributing to the decreased liver fibrosis and angiogenesis. Interesting, HIF-1 is an important molecular in gene upstream of PlGF and VEGF (25, 45). Moreover, inflammatory cell infiltration has often been linked to angiogenesis (40, 44). It has been previously shown that PlGF activated and attracted macrophages, which are capable of releasing angiogenic and lymphangenic molecules mediating angiogenesis (17, 39).

Therefore, these findings suggest that PlGF is involved in hepatic macrophage infiltration and Kupffer cell activation during chronic liver injury, leading to liver fibrogenesis and promoting hepatic angiogenesis, along with HSCs activation. Our results also supported the notion that selective inactivation of Kupffer cells represents a potential mechanism aimed to disrupt the sequence of events leading to liver injury (31–35). However, it is important to mention that macrophages have divergent functions in fibrogenesis and specific populations also promote the resolution of fibrosis in liver through enhanced ECM degradation (3–6, 31, 46). This highlights that further exploring the difference activities of these various macrophages phenotypes during liver fibrosis and resolution of fibrosis are of importance therapeutically.

Mechanically, PlGF specifically binds VEGFR1 and not VEGFR2 (17, 18), activation of VEGFR1 in macrophages by VEGF or by PlGF, contributes to the exacerbation of certain pathophysiological conditions such as inflammation (17, 22, 37). Moreover, PlGF may induces VEGF release from mononuclear cells, and the binding of PlGF to VEGFR1 leads to intermolecular crosstalk between VEGFR1 and VEGFR2, which amplifies VEGFR2 signaling and consequently enhances VEGF-driven response (37, 47, 48). Therefore, the inhibition of PlGF also could suppress both VEGF-driven inflammation and angiogenesis (22, 38, 47, 48). This concept is supported by our present *in vivo* and *in vitro* studies, indicating that VEGFR1 is overexpressed on macrophages upon injury or rPlGF challenge *in vitro*; and PlGF promotes the migration and activation of macrophages into fibrotic liver dependent on VEGFR1 (Figure 8). Since blocking PlGF/VEGFR1 signaling axis significantly inhibits macrophages migration and reduces inflammatory gene expression *in vitro* (Figure 9). Recent studies also demonstrated that the PlGF/VEGFR1 signaling axis was involved in cancer-associated angiogenesis (17, 18, 38). Taken together, these observations strongly suggest that either PlGF or VEGFR1 inhibition can provide therapeutic benefit.

However, it is important to mention that our study has some limitations. Firstly, we examined the effect of PlGF on macrophages recruitment and activation in liver fibrosis by siRNA *in vivo*, as other cells in fibrotic liver, such as EC and HSC, also expression PlGF (Figures 1B,C), therefore, this no cell-specific siRNA delivery may also affect those cells and mediated in liver fibrosis. Second, it is worth remembering that VEGFR1 is also expressed on activated HSCs and vascular ECs in fibrotic livers (Figure 8A), studies focusing on the role of VEGFR1 in these cells should provide more insight into the pathogenesis of fibrosis-associated angiogenesis (45). Third, given that NRP-1 is a coreceptor of PlGF, the effect of PlGF on macrophages may also be involved in NRP-1. Finally, although this injection route delivers siRNA preferentially targeted to liver, this is a challenging process and it is necessary to administer PlGF siRNA repeatedly for the continuous knockdown of PlGF mRNA *in vivo* in order to prevent the progression of hepatic fibrosis. Therefore, further studies on the current topic will need to be undertaken.

In conclusion, our study provides evidence that PlGF mediates the pathogenesis in liver inflammation, angiogenesis, and fibrosis. PlGF is a multitasking cytokine in its ability to promote the recruitment macrophages to the liver and to induce macrophages activation during liver injury and fibrosis in BDL mice. Based on these scientific considerations, inhibiting the PlGF signaling could provide a novel therapeutic target for chronic liver diseases.

## Ethics Statement

The experimental protocol was performed in accordance with the guiding principles for the care and use of laboratory animals approved by the Fudan University Animal Care Committee and all animals received humane care.

## Author Contributions

XL and CT conceived the study and wrote the manuscript; XL and CT contributed to the work designing, performing, analyzing, and interpreting data from all the experiments; QY, QJ, YZhou, YZou, and SZ participated in the design, acquisition, analysis, and interpretation of data; ZL and XL carried out the surgery and all the *in vivo* animal experiments; CT and XL interpreted the data and finalized the article. All authors have critically revised and approved the final manuscript and agreed to be accountable for all aspects of the work.

## Conflict of Interest Statement

The authors declare that the research was conducted in the absence of any commercial or financial relationships that could be construed as a potential conflict of interest.
