# Supplementary material for: Placental Growth Factor Contributes to Liver Inflammation, Angiogenesis, Fibrosis in Mice by Promoting Hepatic Macrophage Recruitment and Activation
Source: Front Immunol. 2017 Jul 11;8:801. doi: 10.3389/fimmu.2017.00801 (PMC5504098; doi:10.3389/fimmu.2017.00801)
Supplement: Supplementary file 1 [file presentation_1.pdf]

<u>Supplementary information accompanies this paper</u>

Placental growth factor contributes to liver inflammation, angiogenesis, fibrosis in mice by promoting hepatic macrophage recruitment and activation

Xi Li[1], Qianwen Jin[2,3], Qunyan Yao[2,3], Yi Zhou[2,3], Yanting Zou[2,3], Zheng Li[4], Shuncai Zhang[2,3], Chuantao Tu[2,3*]

Supplementary S1: Experimental Procedures

Immunohistochemistry

The liver tissue sections were deparaffinized with xylene and rehydrated with graded ethanol. Antigen retrieval was performed by boiling the sections in citrate buffer for 10 min. The sections were stained according to routine immunohistochemistry procedures and visualized by means of a Vectastain ABC kit (Vector Laboratories, Inc., Burlingame, CA, USA). Slides were blocked with 5% goat serum and then incubated with primary antibodies overnight at 4℃ at the following concentrations: (1) anti-α-SMA antibody (1:100); (2) anti-Desmin antibody (1:100); (3)anti-F4/80 antibody (1:100); (4)anti-CD68 antibody (1:100); (5)anti-Ly6C antibody (1:100); (6) anti-TLR4 antibody (1:150); (7) anti-TLR9 antibody (1:150); (8) anti-HIF-1α antibody (1:150); (9) anti-CD31(1:100); (10) anti-PlGF (1:100); (11) anti-Collagen III (1:100). All antibodies were diluted in Tris-buffered saline (TBS) containing 3% BSA. Negative-control antibodies consisted of species-matched and where appropriate, immunoglobulin G (IgG) subclass-matched Ig fractions, used at the same dilution as the secondary antibodies. The sections were subsequently washed with

TBST and incubated with horseradish peroxidase (HRP)-conjugated goat anti-rabbit/mouse/rat secondary antibodies, followed by incubation for 5 to 10 min with 3, 3′-diaminobenzidine tetrachloride and visualization of specific staining by light microscopy. Images were taken under a high-power field with a Leica DC500.

siRNA Sequences in vivo study

In Vivo pre-designed PlGF siRNA and in vivo non-targeting control (NTC) siRNA were from Life Technologies (Carlsbad, CA, USA). The sequences of siRNA as following: PlGF siRNA 5’-UCAGUGCCUUAAAACAGAATT-3’；NTC siRNA 5’-AATTCTCCGAACGTGTCACGT-3’.

Supplementary Table 1. Primer sequences used in this study

Target gene	Forward primers (5'-3')	Reverse primers (5'-3')
**PlGF**	AACACAAGAAGCCTCCTAC	CATTCACAGAGCACATCCT
**Collagen 1α1**	TGACTGGAAGAGCGGAGAGT	AGACGGCTGAGTAGGGAACA
**Collagen 3α1**	CCTTCTACACCTGCTCCT	CTTCCTGACTCTCCATCCT
**α-SMA**	AGAACACGGCATCATCAC	GCAGTAGTCACGAAGGAAT
**DESMIN**	CCTACACCTGCGAGATTG	ATCATCACCGTCTTCTTGG
**CD31**	ACAGAGCCAGCAGTATGA	AATGACAACCACCGCAAT
**vWF**	ATGGAGATGGCAGTGGAT	TGGCAGATGGTATGGAATG
**HIF-1α**	CTGCCACCACTGATGAAT	TGCCACTGTATGCTGATG
**F4/80**	TCTGGGGAGCTTACGATGGA	GAATCCCGCAATGATGGCAC
**CD68**	GGGGCTCTTGGGAACTACAC	GTACCGTCACAACCTCCCTG
**Ly6c**	ATTGAGACTTCCTGCCCAGC	GATCCCTGATTGGCACACCA
**TNFα**	GACGTGGAACTGGCAGAAGA	ACTGATGAGAGGGAGGCCAT
**IL-1β**	GTGCAAGTGTCTGAAGCAGC	CAAAGGTTTGGAAGCAGCCC
**MCP-1**	AGCCAACTCTCACTGAAGCC	GGACCCATTCCTTCTTGGGG
**TLR4**	CAGAACAATAGAAGAGGAAGAC	GGCACTAACCACATAGAGAA
**TLR9**	CAGAAGCCACCCCTGAAGAG	GGCAGGAACTGAGAGCCATT
**CXCL10**	GATGGATGGACAGCAGAG	GGAAGATGGTGGTTAAGTTC
**ICAM-1**	CAGTGAGGAGGTGAATGTATA	GATGTGGAGGAGCAGAGA
**VCAM-1**	TTGACATCTCCCCCGGATCT	AACACAAGCGTGGATTTGGC
**VEGFR1**	GGCAGACCAATACAATCCTA	AGCGAGCAGACTTCAATG
**GAPDH**	TCTCCTGCGACTTCAACA	TGTAGCCGTATTCATTGTCA

Supplementary Figure S1

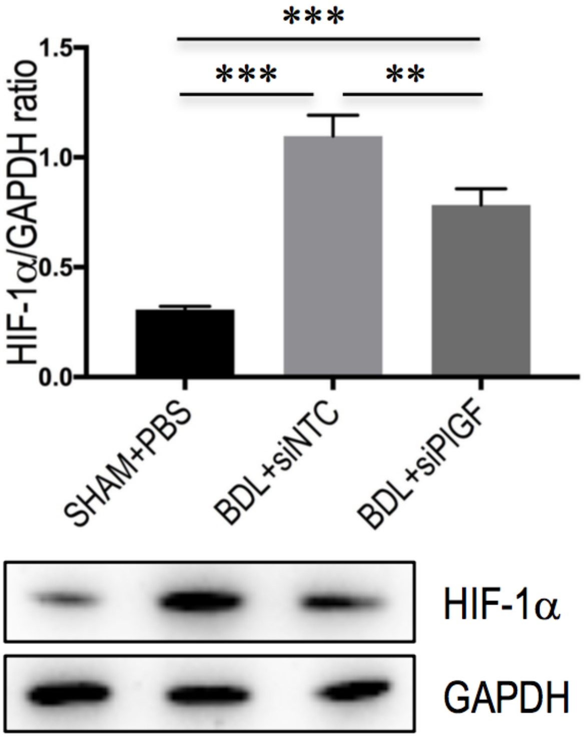

Figure S1. Western blot analysis of hepatic HIF-1α protein expression, with results normalized relative to the expression of GAPDH ($n = 3$). $^{**}P < 0.01$; $^{***}P < 0.001$.

Supplementary Figure S2

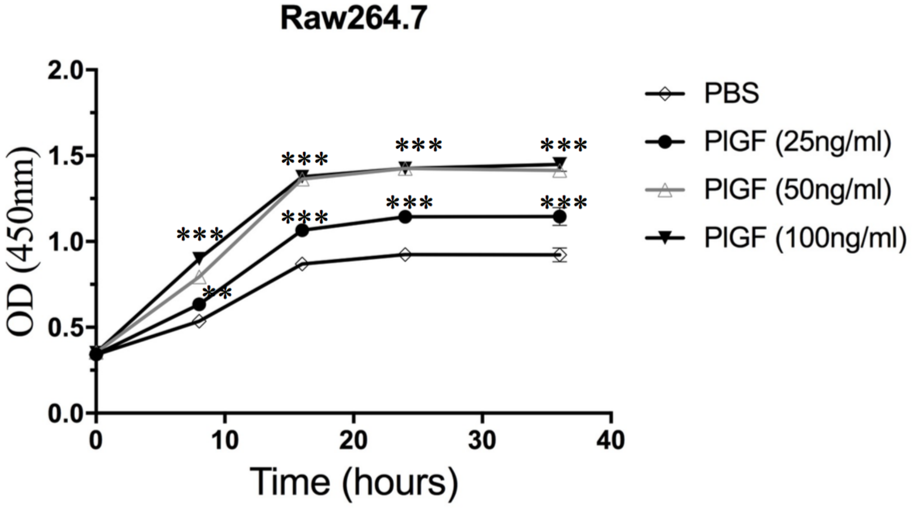

Figure S2. The effect of recombinant PlGF (rPlGF) on RAW 264.7 cell line proliferation was determined using by Cell Counting Kit-8 (CCK-8) assay. RAW 264.7 Cells were cultivated with different dosage mouse rPlGF (0, 25 ng/ml, 50 ng/ml, 100 ng/ml) for different time (0, 8, 16, 24, 36 hours). **P < 0.01, ***P< 0.001 compared with mimics control (PBS). CCK-8 assay was from Yeasen Biotech Company (Shanghai, China) according to the manufacturer's instructions.
